# Cribriform Brain Unraveling Virchow-Robin Space Dilation

**DOI:** 10.7759/cureus.80942

**Published:** 2025-03-21

**Authors:** Hamza Retal, Mohamed Khalil Khabet, Chiara Mabiglia, Anis Soualili, Redouane Kadi

**Affiliations:** 1 Radiology Department, Helora University Hospital, Nivelles-Tubize, BEL; 2 Radiology Department, Erasmus Hospital-Brussels, Brussels, BEL

**Keywords:** brain mri, cystic cerebral lesions, neurodegenerative diseases, neuroradiology, neuroradiology, neuroradiology, neuroradiology, virchow robin spaces

## Abstract

Virchow-Robin spaces (VRS) are perivascular compartments in the brain lined by the pia mater, surrounding penetrating arteries and veins. They play a crucial role in interstitial fluid drainage within the glymphatic system and may contribute to immune responses. Typically small and asymptomatic, these spaces can sometimes enlarge, forming dilated Virchow-Robin spaces, which are often identified incidentally on MRI, especially in elderly individuals. Their clinical significance has been increasingly recognized due to their potential link with neurodegenerative diseases. We present the case of a 60-year-old retired firefighter who experienced recurrent syncopal episodes along with progressive neurological symptoms, including bradykinesia and memory impairment. Brain MRI revealed extensive cystic dilation of periventricular spaces, predominantly in the lenticulostriate region, characteristic of dilated VRS. This case underscores the importance of understanding the mechanisms underlying VRS dilation, distinguishing it from other brain lesions such as lacunar infarcts, neoplasms, and infectious diseases, and highlights the role of MRI in diagnosis. Additionally, we review the literature on dilated VRS and its potential implications in neurodegenerative diseases, including Alzheimer’s disease and chronic traumatic encephalopathy (CTE).

## Introduction

Virchow-Robin spaces (VRS) are perivascular compartments surrounding penetrating arteries and veins in the brain, lined by pia mater cells [1]. These spaces facilitate interstitial fluid drainage, contribute to intracranial pressure regulation, and may have immune-related functions [2]. While they are present in all age groups, they can occasionally enlarge, particularly in the elderly, a condition referred to as dilated VRS.

Some studies have reported a prevalence of 2% for dilated VRS, with the highest occurrence in individuals aged 60 to 79 years and a slight female predominance. They are most frequently located in the basal ganglia and white matter, with type II VRS being the most common. The majority of cases exhibit grade 1 dilation, with only a small percentage reaching significant enlargement. Dilated VRS are often incidental findings but have been associated with cortical atrophy, leukoaraiosis, and microvascular disease [3].

Clinically, dilated VRS are usually asymptomatic but may present with headaches, dizziness, cognitive impairment, or, in rare cases, obstructive hydrocephalus [3]. Their differential diagnosis includes lacunar infarcts, cystic tumors, and infectious diseases such as cryptococcosis and neurocysticercosis. MRI remains the gold standard for differentiation, as dilated VRS appear isointense to cerebrospinal fluid on all sequences, without diffusion restriction or enhancement.

Although dilated VRS are generally benign, their presence may correlate with small vessel disease and neurodegenerative disorders or, in rare cases, lead to hemorrhagic complications. As their clinical relevance continues to be explored, further research is warranted to better understand their implications for neurological health [3].

This report presents a case of extensive dilated VRS, emphasizing the importance of MRI in diagnosis and distinguishing it from other brain lesions. As the clinical relevance of dilated VRS continues to grow, their potential link with neurodegenerative diseases warrants further investigation into their underlying pathophysiology.

## Case presentation

A 60-year-old retired firefighter with a 35-pack-year history of active smoking over 35 years, but no other significant medical conditions, presented to the emergency department due to recurrent syncopal episodes preceded by vertigo. These episodes resulted in traumatic falls but were not followed by postictal confusion.

Upon neurological evaluation, the patient exhibited bradykinesia and chronic memory impairment, which had progressively worsened over several years. Vertigo and syncope were not present during consultation and hospitalization but had emerged one month prior, with two episodes occurring before the consultation. Neurological examination revealed no focal deficits, meningeal signs, or nystagmus. Neuropsychological testing identified impaired working and episodic memory, reduced mental flexibility, and mild visuoconstructive deficits using the Montreal Cognitive Assessment and the Trail Making Test (TMT) A and B. Blood pressure measurement revealed slight hypertension. Cardiovascular assessment, including transthoracic echocardiography, Holter ECG, carotid ultrasound, and electromyography, was unremarkable, ruling out cardiac or peripheral neurological causes.

**Figure 1:**
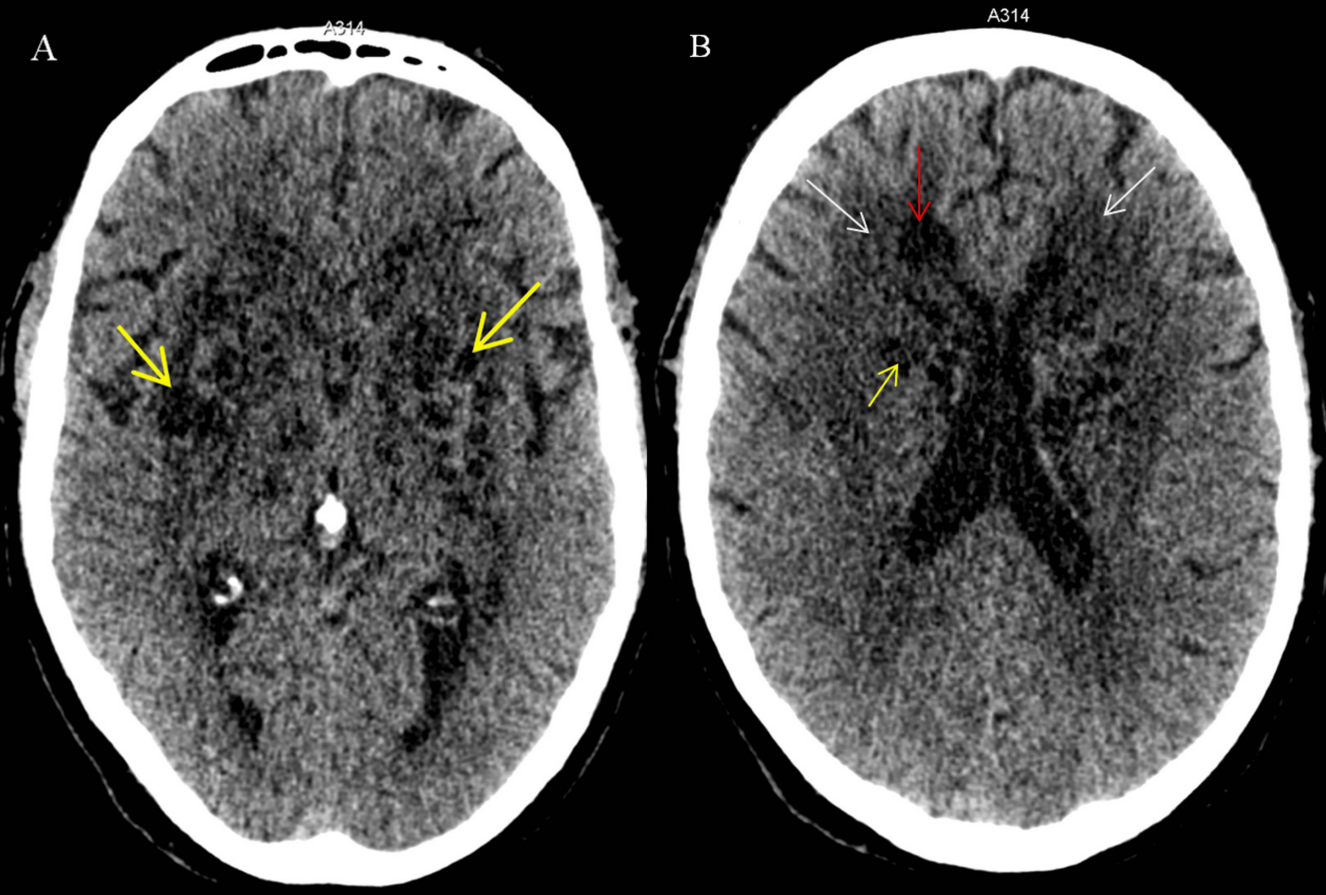
Axial brain CT scan without contrast injection (A) A bubbly appearance due to multiple bilateral cystic lesions in the basal ganglia, predominantly affecting the lenticulostriate region (yellow arrows). (B) Increased hypodensity of the periventricular white matter (white arrow), along with a unilateral hypodense lesion near the right frontal horn of the lateral ventricle in the right frontal lobe (red arrow), in association with multiple cystic lesions within the lenticulostriate region (yellow arrow).

A non-contrast cerebral CT scan revealed multiple extensive hypodense cystic lesions in the basal ganglia and periventricular region, predominantly in the lenticulostriate area, without mass effect (Figure 1).

**Figure 2:**
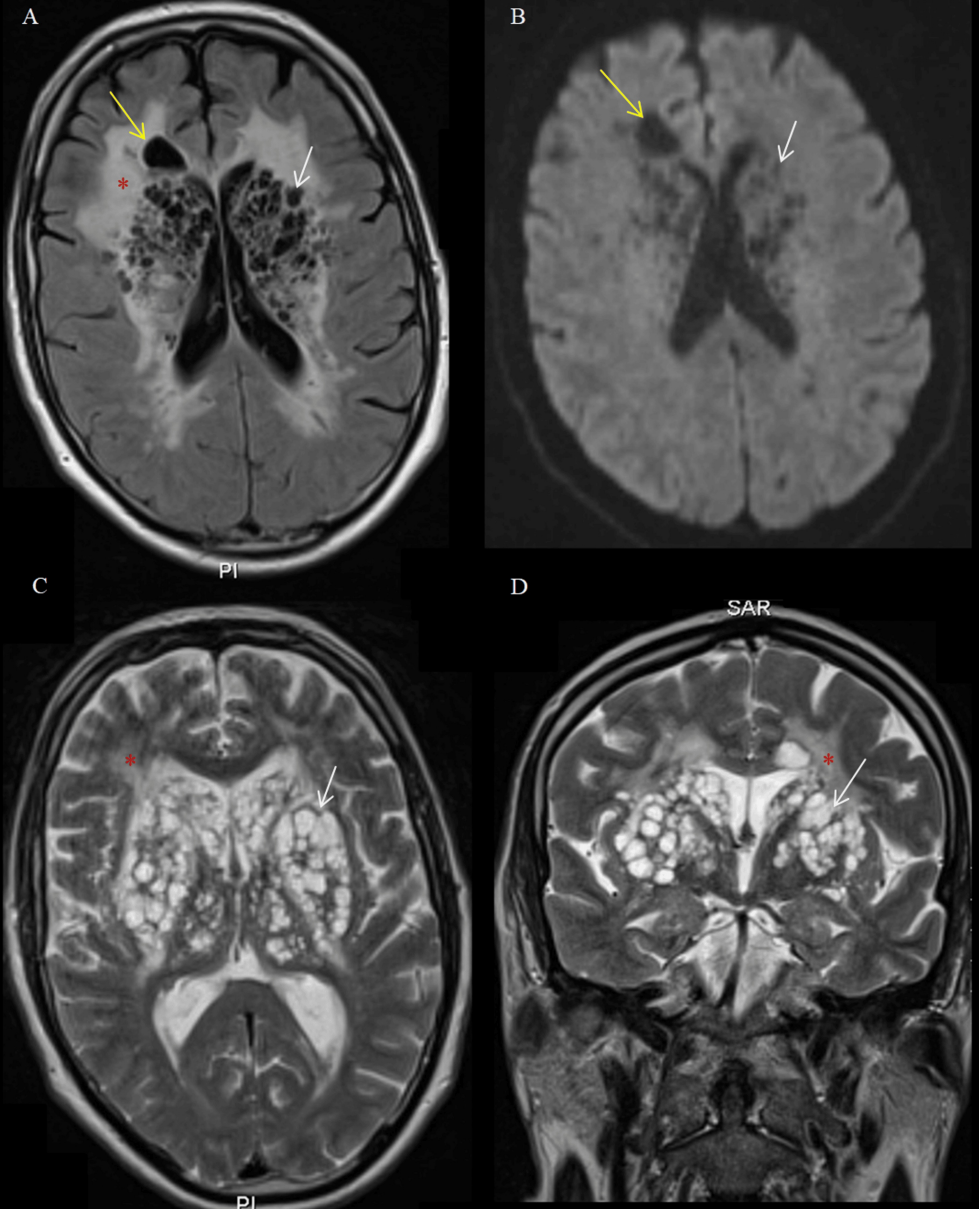
Brain MRI showing bubbly-like cystic lesions, Type I Virchow-Robin space (VRS) dilation, and severe leukoencephalopathy (Fazekas grade 3) (A) Axial FLAIR sequence revealing hypointense CSF-like cystic lesions in the lenticulostriate region (white arrow) and right frontal area (yellow arrow), in association with diffuse periventricular leukoencephalopathy presenting a marked hyperintense signal (red asterisk). (B) DWI sequence demonstrating the absence of diffusion restriction within the cystic lesions in the lenticulostriate region (white arrow) and the right frontal region (yellow arrow). (C) and (D) T2-weighted MRI slices in axial (C) and coronal (D) views demonstrating the corresponding cystic lesions (white arrows) and hyperintense leukoencephalopathy (red asterisk).

There was no evidence of subdural hematoma or hydrocephalus. Subsequent brain MRI showed multiple bilateral and symmetric cystic lesions in the basal ganglia and frontal region, with a CSF-like signal, hyperintense on T2-weighted images, hypointense on T1, and a null signal on FLAIR, without diffusion restriction (Figure 2).

**Figure 3:**
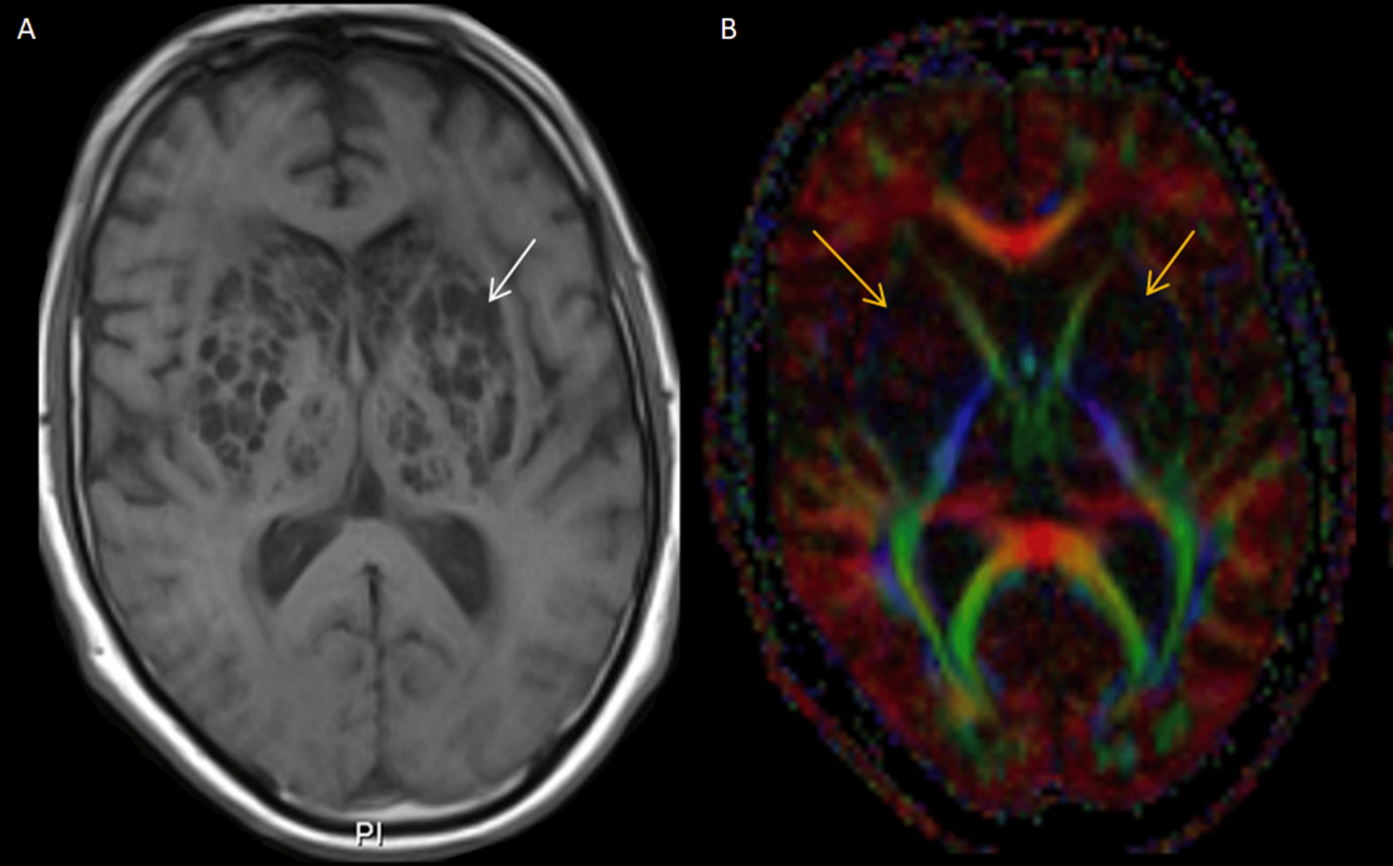
Brain MRI of the cystic lesions (A) Axial post-gadolinium T1-weighted sequence showing no contrast enhancement of the cystic lesions (white arrow). (B) Diffusion tensor imaging (DTI) tractography illustrating the expected organization of white matter tracts within the internal capsule, with no evidence of disruption or mass effect (yellow arrows).

These findings were associated with severe leukoencephalopathy (Fazekas 3), but no cortical abnormalities, mass effect, or abnormal enhancement after gadolinium administration (Figure 3).

**Figure 4:**
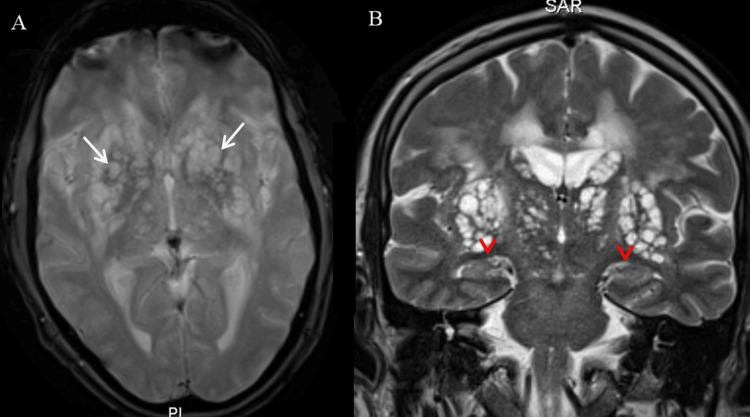
Brain MRI sequences (A) Axial T2*-weighted gradient echo (T2*) image displaying dilated Virchow-Robin spaces (VRS) within the basal ganglia (white arrows), with no hemorrhagic signs or associated magnetic susceptibility artifact. (B) Coronal T2-weighted image at the level of the hippocampal structures (red arrowheads), showing normal hippocampal volume and signal intensity, with no signs of atrophy or hippocampal lesion. The temporal horns of the lateral ventricles remain within normal limits.

There were no signs of cortical or hippocampal atrophy, parietotemporal thinning, or ventricular enlargement suggestive of neurodegeneration. The medial temporal structures were intact, with no evidence of amyloid-related changes or frontotemporal degeneration (Figure 4).

A broad infectious workup was conducted due to concerns about cryptococcosis, a common mimic of dilated VRS in immunosuppressed individuals. In neurocryptococcosis, dilated perivascular spaces can coalesce into "soap bubble" pseudocysts with variable fluid-attenuated inversion recovery (FLAIR) signal, low-to-intermediate T1-weighted (T1) signal, and high T2-weighted (T2) signal, and may show restricted diffusion, whereas dilated VRS always exhibit low FLAIR signal and no enhancement. No immunodepression or cryptococcal antigen was found in cerebrospinal fluid (CSF). Serological tests for syphilis, Borrelia, human immunodeficiency virus (HIV), herpes simplex virus type 1 (HSV-1), herpes simplex virus type 2 (HSV-2), varicella-zoster virus (VZV), cytomegalovirus (CMV), and Epstein-Barr virus (EBV) were negative. Lumbar puncture revealed normal CSF parameters, with polymerase chain reaction (PCR) testing for viral infections also yielding negative results. Given the absence of gliotic changes and the bilateral symmetric nature of the lesions, Type I dilated Virchow-Robin spaces (DVRS) was the most plausible diagnosis. The presence of marked leukoencephalopathy on MRI suggested that the patient’s neurological symptoms were more likely due to vascular leukoencephalopathy and aging-related changes rather than VRS dilation alone. During one-year follow-up, the patient remained clinically stable, with spontaneous resolution of syncopal episodes and no further deterioration in memory.

## Discussion

First described by Rudolf Virchow and Charles Philippe Robin, VRS are fluid-filled perivascular structures that accompany penetrating arteries and veins but do not communicate with the subarachnoid space [4]. Incidentally found in approximately 2% of the population, their prevalence increases with age, potentially serving as an indirect marker of brain tissue loss. When abnormally enlarged (>2 mm), they are frequently detected on MRI, particularly in the basal ganglia, thalamus, midbrain, cerebellum, and corpus callosum [5,6]. Though usually asymptomatic, excessive dilation may exert a mass effect, potentially leading to obstructive hydrocephalus [5,6].

VRS are integral to the glymphatic system, playing a key role in interstitial fluid drainage and possibly contributing to immune regulation [4,7]. Some studies suggest that VRS function as part of a "protolymphatic" system, mediating fluid exchange between CSF and interstitial fluid to aid in metabolic waste clearance [7]. Excessive dilation of VRS, particularly in cases of giant tumefactive perivascular spaces (GTPVS, ≥1.5 cm), may resemble lacunar infarcts, cystic neoplasms, or infectious cysts, necessitating precise differentiation [8]. On MRI, VRS appear isointense to CSF on T1- and T2-weighted images, are suppressed on FLAIR, and do not exhibit restricted diffusion, calcification, hemorrhage, or contrast enhancement [1]. However, hemorrhage has been described as a rare presentation in some case reports as a post-traumatic complication [9]. In contrast, vascular infarcts typically display a hyperintense gliotic rim on FLAIR, allowing distinction. The most similar appearance to our findings was cryptococcosis, which typically causes bubbly cystic lesions in the brain, as reported in some literature cases [10].

Additional imaging techniques, including high-resolution MRI, contrast-enhanced sequences, TOF angiography, and diffusion tensor imaging (DTI), provide further diagnostic accuracy by assessing fiber integrity and detecting possible fiber compression or distortion due to VRS expansion and vascular components (perforating arteries) within VRS [5,11]. VRS are classified into three primary types based on location: Type I (basal ganglia, lenticulostriate arteries), Type II (cortical gray matter, perforating medullary arteries), and Type III (midbrain), with a Type IV variant described in the anterior temporal operculum [12]. In some instances, GTPVS in the midbrain may cause a mass effect on the aqueduct of Sylvius, leading to hydrocephalus [5].

Although DVRS are usually incidental, they have been associated with several neurological disorders, including Alzheimer’s disease, chronic traumatic encephalopathy (CTE), traumatic brain injury (TBI), and cerebrovascular disease. A study of 79 healthy men found that widened VRS correlated with white matter lesions but did not independently affect cognitive function, suggesting that they may reflect localized atrophy rather than direct neurodegeneration [13]. Some reports indicate a potential role in epilepsy, though the precise mechanism remains unclear. VRS dysfunction may also contribute to impaired glymphatic clearance, resulting in metabolic waste accumulation, neuroinflammation, and neurodegeneration, particularly in TBI and CTE. Furthermore, GTPVS has been frequently observed in diabetic patients, possibly due to altered glymphatic function and molecular waste buildup [5].

Establishing a direct association between the patient’s cognitive decline and dilated VRS is challenging, particularly in the context of severe leukoaraiosis, a well-recognized contributor to cognitive impairment. While dilated VRS may play a role, the extensive white matter changes suggest an underlying vascular pathology as the predominant cause of the patient’s symptoms. Additionally, studies exploring the association between dilated VRS and dementia are highly heterogeneous, primarily due to the limited number of available studies and the variety of study designs employed. These challenges highlight the complexity of distinguishing vascular from glymphatic contributions to cognitive decline. Given the role of the glymphatic system in brain waste clearance, its dysfunction might contribute to cognitive impairment, particularly in patients with dilated VRS. Recent advances in MRI-based imaging could provide new insights into whether glymphatic dysfunction plays a role in neurodegeneration. Further research is needed to clarify these mechanisms and their clinical implications, potentially differentiating between vascular and glymphatic contributions to cognitive decline [14,15].

## Conclusions

VRS are normal perivascular structures, but their dilation may serve as an indicator of neurovascular pathology. Their role in glymphatic function and possible association with neurodegeneration, epilepsy, and cerebrovascular disease necessitate further research. MRI remains the key diagnostic tool for identifying dilated VRS, enabling accurate differentiation from pathological cystic and ischemic lesions, thereby reducing the risk of misdiagnosis and avoiding unnecessary investigations or treatments.
